# Species–area relationships and biodiversity loss in fragmented landscapes

**DOI:** 10.1111/ele.12943

**Published:** 2018-03-30

**Authors:** Ryan A. Chisholm, Felix Lim, Yi Shuen Yeoh, Wei Wei Seah, Richard Condit, James Rosindell

**Affiliations:** ^1^ Department of Biological Sciences Faculty of Science National University of Singapore 14 Science Drive 4 Singapore 117543 Singapore; ^2^ Department of Animal and Plant Sciences University of Sheffield Sheffield S10 2TN UK; ^3^ Department of Zoology New Radcliffe House Radcliffe Observatory Quarter Woodstock Rd Oxford OX2 6GG UK; ^4^ Herbarium, Singapore Botanic Gardens National Parks Board 1 Cluny Road Singapore 259569 Singapore; ^5^ Smithsonian Tropical Research Institute P.O. Box 0843‐03092 Balboa Ancón Panama; ^6^ Department of Life Sciences Imperial College London Silwood Park campus, Buckhurst Road Ascot Berkshire SL5 7PY UK

**Keywords:** Deforestation, fragmentation, habitat loss, rescaling, scaling, species–area curve

## Abstract

To estimate species loss from habitat destruction, ecologists typically use species–area relationships, but this approach neglects the spatial pattern of habitat fragmentation. Here, we provide new, easily applied, analytical methods that place upper and lower bounds on immediate species loss at any spatial scale and for any spatial pattern of habitat loss. Our formulas are expressed in terms of what we name the ‘Preston function’, which describes triphasic species–area relationships for contiguous regions. We apply our method to case studies of deforestation and tropical tree species loss at three different scales: a 50 ha forest plot in Panama, the tropical city‐state of Singapore and the Brazilian Amazon. Our results show that immediate species loss is somewhat insensitive to fragmentation pattern at small scales but highly sensitive at larger scales: predicted species loss in the Amazon varies by a factor of 16 across different spatial structures of habitat loss.

## Introduction

To estimate the number of species lost as a result of habitat destruction, ecologists typically turn to species–area relationships (SARs), which take habitat area as an input and give species richness as an output. This approach has several limitations (Lewis 2006). First, it ignores the dependence of species loss on the time elapsed since habitat destruction occurred. Here, we study the shortest timescale possible – immediate species loss – which is a prerequisite to understanding more complex long‐term problems where losses from extinction debt repayment are added to immediate losses. Second, while traditional SARs assume that species persist only in remnant habitat areas, in practice some species may persist in non‐habitat areas too. Previous studies have tackled this second problem (Pereira & Daily 2006). Here, we retain the assumption that species live only inside habitat areas in order to focus on the third major limitation of the traditional species–area approach: the assumption that total habitat area is the only spatial parameter of importance. In practice, fragmentation and spatial structure – of both habitat loss and species distributions – are also critical.

If one has complete information on the spatial distribution of species, including their abundances, one can compute the number of species and the number of endemics in an arbitrary region of habitat; the number of endemics gives the number of species immediately lost when that habitat is destroyed (Kinzig & Harte 2000; He & Legendre 2002; Green & Ostling 2003; He & Hubbell 2011, 2013; Pereira *et al*. 2012). In practice, however, such complete information is usually lacking. The traditional fallback approach has been to estimate species loss from an SAR by comparing the species richness calculated from an initial area to that from a reduced area. But this is only accurate if one of two restrictive conditions is satisfied: either species are distributed randomly in space, or the SAR is constructed such that the spatial pattern of habitat considered is exactly opposite (complementary) to the spatial structure of *destroyed* habitat in the target system (He & Hubbell 2013). These considerations are routinely ignored in practice, with the typical approach being to parameterise an SAR with data from contiguous patches of habitat and apply it to a fragmented target system.

The above concerns apply to any SAR, that is, any function that expresses species richness solely as a function of habitat area. The most common specific approach is to use a power‐law function of area (Arrhenius 1921) with exponent in the range 0.2–0.3. But these typical exponents come from fits to data from contiguous habitat patches (e.g. Storch *et al*. 2012), which are not complementary to the complex and usually fragmented patterns of real world habitat destruction and thus not suitable for predicting species loss. Further problems with the power‐law SAR are that it is phenomenological, rather than mechanistic, and that it ignores scale‐dependent variation in the power‐law exponent observed in empirical data (Harte *et al*. 2009).

Beyond the power‐law SAR, more sophisticated mechanistic SAR models have been developed (Durrett & Levin 1996; Rosindell & Cornell 2007; O'Dwyer & Green 2010; Grilli *et al*. 2012). Such models accurately characterise the contiguous SARs seen in nature, with scale‐dependent power‐law exponents and an overall triphasic shape (Preston 1960; Plotkin *et al*. 2000; Storch *et al*. 2012), but share the one key limitation with the power‐law SAR when it comes to predicting species loss from habitat destruction: they implicitly assume contiguous habitat.

Several studies have pushed the envelope and studied fragmented SARs, which allow one to predict how species loss is affected by the spatial pattern of habitat fragmentation, not just total habitat area loss. These studies have typically used phenomenological extensions of the power‐law model (Kinzig & Harte 2000; He & Hubbell 2011; Pereira *et al*. 2012) or spatial simulations (Campos *et al*. 2012; Hanski *et al*. 2013; Keil *et al*. 2015), and have mostly found that fragmented habitat destruction causes less immediate species loss than does contiguous habitat destruction, because fragmented habitat arrangements preserve more of a landscape's beta diversity. Indeed, simulations suggest that immediate species loss is lowest when the landscape is cleared randomly (Claudino *et al*. 2015). Simulations also suggest that fragmented SARs exhibit a qualitatively similar triphasic shape to contiguous SARs (Campos *et al*. 2013). But to establish the generality of these conclusions and to facilitate species loss calculations at spatial scales where simulations are infeasible, we require analytical results from mechanistic models, which have thus far been elusive. The only such result we know of is for when habitat destruction is spatially random and each species’ spatial distribution follows a finite version of a negative binomial distribution (Zillio & He 2010; He & Hubbell 2011).

Here, we make substantial progress towards the problem of analytically calculating immediate species loss in fragmented landscapes. Our analyses are based on spatially explicit communities generated by a neutral model (Hubbell 2001; O'Dwyer & Chisholm 2013). Neutral theory is not the focus of our study; we apply it here because it generates artificial communities that look similar to natural ones while being analytically tractable across multiple scales. Our results place upper and lower bounds on immediate species loss at any spatial scale and for any spatial pattern of habitat destruction.

## Methods

### Clearing patterns

**Figure 1:**
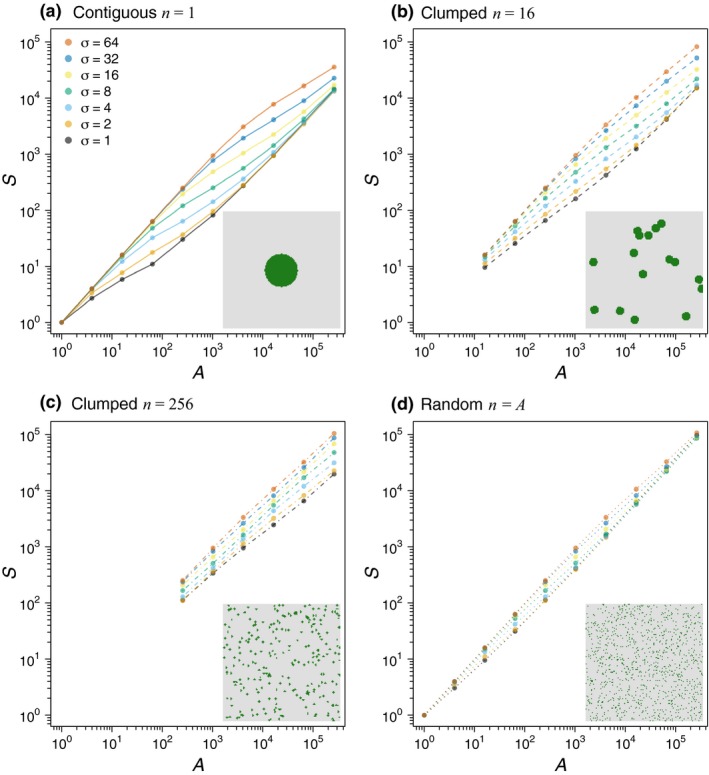
Insets: Types of habitat clearing considered in this study. Green pixels indicate habitat; grey pixels indicate cleared areas. Panel a shows classic contiguous clearing (*n* = 1); panels b and c show clumped clearing with *n* = 16 and *n* = 256 clumps respectively; panel d shows random clearing (*n* = *A*). Main panels: SARs resulting from the four types of habitat clearing shown in insets, for a range of values of landscape extent *A*_*max*_ and total habitat area *A* = *A*_*max*_/16 (thus *A* and *A*_*max*_ vary proportionally on the horizontal axes). The ecological communities to which the clearing maps are applied are spatial neutral communities with speciation rate ν = 0.01 and a range of values for the dispersal standard deviation σ (colours; see legend on panel a).

Empirical habitat destruction typically exhibits complex fractal‐like patterns (Gardner *et al*. 1987; Sugihara & May 1990; With 1997; Hargrove *et al*. 2002; Ewers & Laurance 2006). In theoretical studies, patterns used include fractal structures (With 1997), percolation maps (Campos *et al*. 2012) and distinct clumps of habitat or non‐habitat (He & Hubbell 2011). Some results we derive here apply to all clearing patterns but we focus on three cases: ‘contiguous clearing’, where remaining habitat is a contiguous block (Fig. 1a inset); ‘clumped clearing’, where remaining habitat comprises several clumps that are randomly distributed throughout a landscape (Fig. 1b,c insets); and ‘random clearing’, where the clearing pattern is random at the scale of individual organisms, with no spatial autocorrelation (Fig. 1d inset). Random and contiguous clearing provide upper and lower bounds on species richness, and hence lower and upper bounds on immediate species loss (Claudino *et al*. 2015). Heuristically, this is because remaining habitat under random clearing is sampled uniformly across the landscape, maximising beta diversity, whereas contiguous clearing maximises the spatial autocorrelation among remaining habitat, minimising beta diversity.

To characterise our clearing patterns, we use three parameters: the area of remaining habitat *A*; the total area of the landscape of interest *A*_*max*_ ≥ *A*; and the number of fragments *n*, with *n* = 1 corresponding to contiguous clearing, *n* = *A* to random clearing, and 1 < *n* < *A* to clumped clearing. We constructed the clumped clearing maps by randomly choosing *n* clump centroids on a gridded landscape of area *A*_*max*_, and then marking as habitat the *A* grid cells nearest to centroids.

### Spatial neutral model

To create artificial and generic communities on which to study the immediate effects of habitat loss, we required a spatially explicit model that produces plausible species distributions and is tractable for large landscape sizes. We chose the spatial neutral model on an infinite two‐dimensional landscape, which comes in two versions: zero‐sum and non‐zero‐sum. In the zero‐sum version (e.g. Rosindell & Cornell 2007), exactly one individual occupies each grid cell. Individuals rain propagules down in a pattern governed by a radially symmetric dispersal kernel with variance σ^2^. In most of the following we assume that the kernel is a bivariate normal distribution, but many of our results generalise to any dispersal kernel with finite variance (σ^2^ < ∞). Individual death follows a Poisson process, and each cell vacated by a death is immediately replaced by a new recruit drawn at random from the propagule rain onto the cell. There is a small but non‐zero probability ν that the new recruit transforms into a new species, that is, ν is the per‐capita probability of point speciation. The non‐zero‐sum version of the model (e.g. O'Dwyer & Cornell 2017) is similar, except that deaths and births are independent processes, positions of individuals are not constrained to a grid, and new species enter the community via an independent Poisson process whose rate parameter ν is constant in time and space and measured per unit area.

Of the two versions of the spatial neutral model, the zero‐sum is generally easier to simulate, and non‐zero‐sum is more tractable analytically. But they yield almost identical results except in some limiting cases, for example, σ^2^ → 0. In particular, they reproduce the triphasic contiguous SAR characteristic of empirical data (Rosindell & Cornell 2007), with the first phase occurring at scales *A* < σ^2^, the second phase at σ^2^ < *A* < σ^2^/ν and the third phase at *A* > σ^2^/ν (O'Dwyer & Green 2010). Here, we draw on coalescence theory to simulate the zero‐sum model (Rosindell *et al*. 2008) and analytical solutions for the non‐zero‐sum model (O'Dwyer & Cornell 2017; also see Appendix 1).

Because we are here concerned with immediate species loss, we solve the spatially explicit neutral model at the dynamic equilibrium on a contiguous landscape, and then subsequently fragment the community and measure immediate species loss (if we were instead investigating long‐term species loss, we would need to find the dynamic equilibrium on the fragmented landscape).

Our results from the neutral model can be applied to non‐neutral systems provided that the constituent species’ spatial distributions are statistically similar to those of neutral species. In such applications, the parameters σ and ν can be chosen to match data on species’ spatial distributions, with σ controlling spatial autocorrelation and ν controlling overall landscape diversity (Condit *et al*. 2002) (see Appendix 2 for details).

### Rescaling approach

We now define functions that relate species richness immediately after habitat clearing to the parameters of the clearing map and the community model. Except where otherwise noted, the sampling area *A*, landscape area *A*_*max*_ and dispersal parameter σ are measured in units such that there is one individual per unit area. For the contiguous‐clearing case, we define $S_{\mathrm{contig}}\left(A,\nu ,\sigma^{2}\right)$, which is a contiguous SAR and depends only on the area *A* of a contiguous habitat region and the two underlying community model parameters. For the random‐clearing case, we define $S_{\mathrm{random}}\left(A_{\max},A,\nu ,\sigma^{2}\right)$, which additionally depends on the underlying landscape area *A*_*max*_. The clumped‐clearing case depends also on *n* and is described by $S_{\mathrm{clump}}\left(n,A_{\max},A,\nu ,\sigma^{2}\right)$.

The following rescaling relationship for $S_{\mathrm{contig}}\left(A,\nu ,\sigma^{2}\right)$ has been derived previously (Rosindell & Cornell 2007) for σ^2^ ≫ 1:

(1) $$\begin{array}{c} S_{\mathrm{contig}}\left(A,\nu ,\sigma^{2}\right)∼\sigma^{2}\Psi \left(\frac{A}{\sigma^{2}},\nu \right) \end{array}$$

for some function Ψ, or, equivalently,

(2) $$\begin{array}{c} S_{\mathrm{contig}}\left(kA,\nu ,k\sigma^{2}\right)∼kS_{\mathrm{contig}}\left(A,\nu ,\sigma^{2}\right) \end{array}$$

for any scalar *k*. Formula (1) reduces the number of effective parameters so that instead of having to understand the three‐parameter function *S*_*contig*_, we need to only understand the simpler two‐parameter function Ψ.

In view of the central importance of the neutral contiguous‐SAR function in ecological theory, we henceforth refer to Ψ as the ‘Preston function’, after Frank Preston who first observed the triphasic SAR in empirical data and understood the basic mechanisms behind it (Preston 1960). Specifically, we define the Preston function to be the SAR for contiguous circular areas in the non‐zero‐sum spatial neutral model with a bivariate normal dispersal kernel (O'Dwyer & Green 2010; O'Dwyer & Cornell 2017). We choose this definition because the non‐zero‐sum model is more amenable to analytical treatment and, anyway, produces almost identical SARs to the zero‐sum model. Abstracting the Preston function away from the main focus of our work enables us to derive rescaling formulas independently of the mathematical study of the Preston function itself (O'Dwyer & Green 2010; Grilli *et al*. 2012; O'Dwyer & Cornell 2017). The combination of the new scaling results we present below and any method to evaluate the Preston function yields analytical approximations of *S*_*contig*_, *S*_*random*_ and *S*_*clump*_ that predict immediate species loss under a range of scenarios at arbitrary spatial scales.

### Case studies

After deriving our rescaling formulas, we applied them to three real species‐loss problems spanning a range of spatial scales. We focused our applications on tropical tree communities, for several reasons: (1) Beta diversity of tropical trees over distances of 0.5–50 km is statistically similar to beta diversity under neutral theory (Condit *et al*. 2002), which suggests that tree species’ spatial distributions at these scales are also statistically similar to those under neutral theory (notwithstanding a current paucity of individual‐mapped data at these scales). (2) Trees are sessile and this simplifies the question of immediate species loss because individuals cannot relocate to escape habitat destruction. (3) Immediate species loss is most relevant to communities in which extinction debt is small or repaid slowly, and in tree communities the latter condition is likely to be satisfied because trees are sedentary and long‐lived (the same is not true for, e.g. vertebrates). (4) There are substantial data available for tropical tree communities.

Our definition of ‘tree’ is a vascular plant that can grow to at least 10 cm diameter‐at‐breast‐height (DBH). For all case studies, we used estimates of tree density and dispersal distance from the well‐studied tropical forests of central Panama: our estimate of tree density was ρ = 42 000 km^−2^ (Hubbell *et al*. 2005); our estimate of dispersal standard deviation was σ = 40.2 m (Condit *et al*. 2002). Although in reality ρ and σ vary across tropical forests, these ballpark estimates serve the purpose of demonstrating our methods and producing approximate estimates of species loss.

For each case study, we first determined from empirical data, the initial area *A*_*max*_, post‐clearing area *A*, and initial species richness *S*_*max*_. The values of *A*_*max*_ and *S*_*max*_ were used as constraints to estimate the speciation rate parameter ν separately for each case study (Appendix 2). Initial species richness, initial area, final area and dispersal distance were thus the only data used to fully parameterise our model: our predictions were emergent from these data and not fitted to the empirical species loss numbers. Next, we used our analytical formulas to estimate the post‐clearing species richness *S*_*contig*_ and *S*_*random*_ under contiguous‐ and random‐clearing scenarios, respectively, and thus the number of endemic species in the cleared areas, $E_{contig^{\prime }}=S_{\max}-S_{\mathrm{contig}}$ and $E_{random^{\prime }}=S_{\max}-S_{\mathrm{random}}$, under the same two scenarios. Species loss under each scenario can then be equated to the number of species that were endemic to the cleared region (He & Hubbell 2013): $S_{loss,contig}=E_{contig^{\prime }}$ and $S_{loss,random}=E_{random^{\prime }}$. These provide upper and lower bounds on immediate species loss under arbitrary clearing scenarios (Claudino *et al*. 2015).

The first case study was at the local scale of an *A*_*max*_ = 50 ha forest plot on Barro Colorado Island in the Panama Canal (Hubbell *et al*. 2005). The number of tree species in the plot in 2010 was *S*_*max*_ = 222. Our clearing scenario here was hypothetical, with area losses of 10–90% explored. We compared these estimates to those from simulated random and contiguous clearing on the actual 50 ha plot spatial census data.

Our second case study was at the regional scale and corresponded to the entire tropical island city‐state of Singapore (Corlett 1992). Prior to 1819, Singapore was almost entirely forested. From 1819 to 1992, Singapore's forested area decreased from approximately $A_{\max}=540\;km^{2}$ to $A=23\;km^{2}$ (Corlett 1992), representing 95.7% forest loss. From herbarium records, we estimated Singapore's tree species richness in 1819 as *S*_*max*_ = 840 and current tree species richness as *S* = 728, implying Δ*S* = 112 extinctions. In addition, we estimated the number of undetected extinctions (species that went extinct before they could be recorded) from a nonparametric statistical method (Chisholm *et al*. 2016) that requires as input only the time series of herbarium records. The method estimated 89 undetected extinctions and a corresponding upper bound on species loss of Δ*S* = 112 + 89 = 201.

Our third case study was at the continental scale and covered the Brazilian Amazon. The true magnitudes of tree species diversity and extinction in the Amazon are highly uncertain. Accordingly, this case study is not an exercise in model validation, but a demonstration of our formulas’ power to make predictions easily and quickly at arbitrarily large spatial scales. We used input parameter values from the optimistic scenario of Hubbell *et al*. (2008): original area *A*_*max*_ = 4 652 400 km^2^, current area *A* = 0.633*A*_*max*_ and original tree species richness *S*_*max*_ = 11 210. We also compared our model's predictions to those of Hubbell *et al*. (2008), who used a more complex spatial simulation approach.

In Appendix 3, we provide R code for reproducing our case study results and for applying our methods more broadly. As input, the R code requires five parameter values: initial habitat area *A*_*max*_, final habitat area *A*, initial species richness *S*_*max*_, density of individuals per unit area ρ and standard deviation of dispersal distance σ. The parameter σ can be estimated either directly from dispersal data (e.g. from seed trap data for plants (Muller‐Landau *et al*. 2008) or telemetry data for animals) or indirectly from data on the spatial distribution of organisms, as done for our case studies here (Condit *et al*. 2002) (Appendix 2). The R code returns estimated species loss under contiguous and random clearing (running time is on the order of milliseconds).

## Results

### Changing spatial scale

Our first theoretical result (Appendix 4) is that rescaling relationships similar to those shown in formula (2) also apply to arbitrary clearing patterns, including clumped clearing and random clearing:

(3) $$\begin{array}{c} S_{\mathrm{clump}}\left(n,\;kA_{\max},kA,\nu ,k\sigma^{2}\right)∼kS_{\mathrm{clump}}\left(n,A_{\max},A,\nu ,\sigma^{2}\right) \end{array}$$

(4) $$\begin{array}{c} S_{\mathrm{random}}\left(kA_{\max},kA,\nu ,k\sigma^{2}\right)∼kS_{\mathrm{random}}\left(A_{\max},A,\nu ,\sigma^{2}\right) \end{array}$$

**Figure 2:**
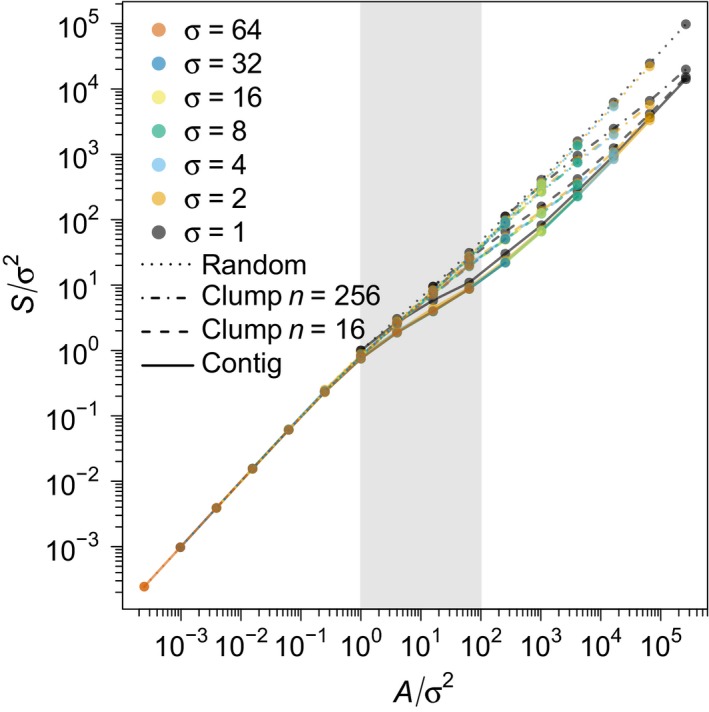
The SARs from Fig. 1 rescaled by 1/σ^2^ on both axes. Solid curves correspond to contiguous clearing (Fig. 1a); dashed and dash‐dotted curves correspond to clumped clearing with *n* = 16 and *n* = 256 clumps respectively (Fig. 1b,c); dotted curves correspond to random clearing (Fig. 1d). As predicted by formulas (2), (3), (4), there is a scaling collapse within each of the four clearing types and the collapse is better for large σ (in this case, for σ ≥ 2). The shaded grey region indicates the second phase of the SAR: σ^2^ < *A* < σ^2^/ν. (See Fig. 3a for a zoomed‐in version of the second and third SAR phases of this graph, without the σ = 1 curve).

**Figure 3:**
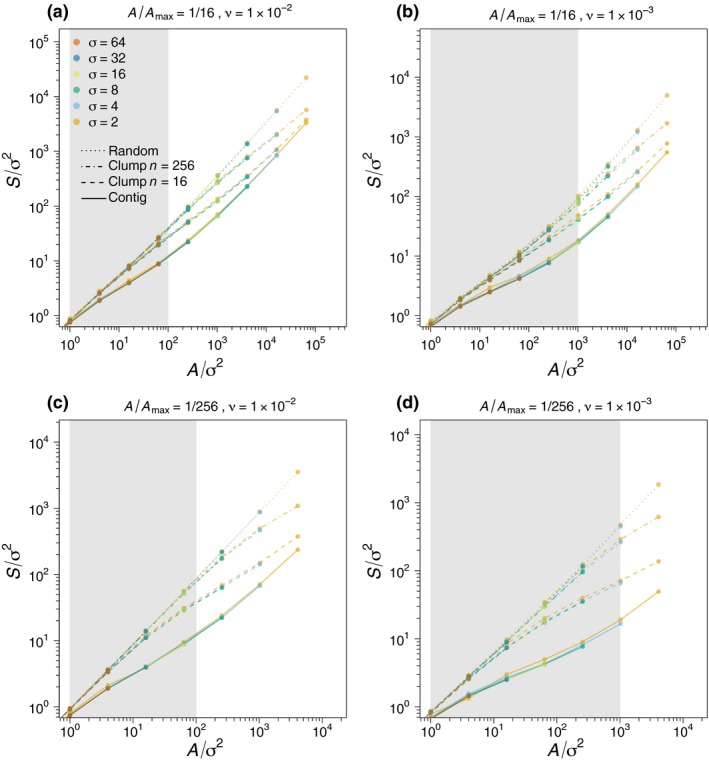
Rescaled SARs as in Fig. 2 but showing only the second and third SAR phases, and also omitting the σ = 1 case (which does not exhibit a good scaling collapse; see Fig. 2). Panel a is for the same parameter values as Fig. 2 (habitat‐to‐landscape area ratio *A*/*A*_*max*_ = 1/16 and speciation rate ν = 0.01); Panel b has a lower speciation rate ν = 0.001; Panels c and d repeat panels a and b with a lower habitat‐to‐landscape area ratio *A*/*A*_*max*_ = 1/256.

**Figure 4:**
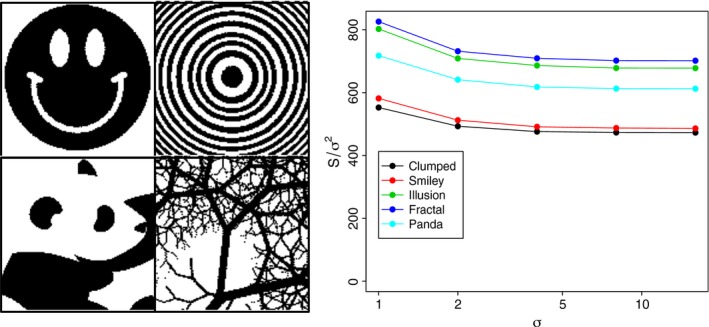
Arbitrary clearing patterns (left) and a test of the hypothesis that the species richness after clearing (*S*) divided by the variance of the dispersal kernel (σ^2^) should be invariant under the spatial rescaling of both the clearing map and σ, provided that σ is not too small (the clearing map is ‘stretched’ equally in all directions proportionately with increasing dispersal distance σ; see Appendix 4 for details). Clearing patterns (from top‐left, moving clockwise) are ‘smiley’, ‘illusion’, ‘fractal’, and ‘panda’. A ‘clumped’ clearing pattern with 16 clumps (Fig. 1b inset) is also included. Each data point shows the mean species richness for the corresponding clearing pattern (colours) applied to 10 replicate communities (speciation rate ν = 0.01, σ values on horizontal axis).

These formulas can be understood intuitively in terms of coalescence processes (Appendix 4). We confirmed them numerically for the clumped clearing case (see Fig. 1b,c and dashed and dash‐dotted curves in Figs 2 and 3), the random clearing case (see Fig. 1d and dotted curves in Figs 2 and 3) and for arbitrary clearing patterns (Fig. 4).

### Relating clumped clearing to other clearing patterns

We also derived theoretical rescaling relationships between qualitatively different types of clearing. Three different results emerged relating a clumped‐clearing pattern (see Fig. 1b,c insets) to a contiguous‐clearing pattern (see Fig. 1a inset). The first result occurs when the mean species range size is small so that any given species is unlikely to appear in more than one clump: *A*_*max*_/*n* ≫ σ^2^/ν. This decouples the clumps from one another so that total species richness is simply the sum of the species richness in each independent clump:

(5) $$\begin{array}{c} S_{\mathrm{clump}}\left(n,A_{\max},A,\nu ,\sigma^{2}\right)∼nS_{\mathrm{contig}}\left(\frac{A}{n},\nu ,\sigma^{2}\right) \end{array}$$

Our second result for clumped clearing occurs under the stricter condition that *A*/*n* ≫ σ^2^/ν so that the clumps themselves become larger than the average species range size. Consequently, there is negligible overlap of species composition between clumps, even if the clumps are repositioned right next to one another. Merging the clumps to form a contiguous‐clearing pattern therefore does not affect species loss and clumped clearing becomes equivalent to contiguous clearing:

(6) $$\begin{array}{c} S_{\mathrm{clump}}\left(n,A_{\max},A,\nu ,\sigma^{2}\right)∼S_{\mathrm{contig}}\left(A,\nu ,\sigma^{2}\right) \end{array}$$

This result is demonstrated in Fig. 3, where the dashed and dash‐dotted curves (clumped clearing) begin to converge to the solid curves (contiguous clearing) as *A*/σ^2^ becomes large, with faster convergence for smaller *n* and larger ν.

Our third result for clumped clearing occurs at the opposite extreme where the average species range size (σ^2^/ν) is large enough to overlap many patches, that is, *A*_*max*_/*n* ≪ σ^2^/ν. In this scenario, species are well mixed at the scale of a few habitat clumps and hence species richness is affected similarly by random and clumped clearing:

(7) $$\begin{array}{c} S_{\mathrm{clump}}\left(n,A_{\max},A,\nu ,\sigma^{2}\right)∼S_{\mathrm{random}}\left(A_{\max},A,\nu ,\sigma^{2}\right) \end{array}$$

We numerically confirmed formula (7) for simulated clumped clearing with *n* = 16, and *n* = 256. In Fig. 3, for instance, we see that clumped clearing converges to random clearing as *A*/σ^2^ (and hence *A*_*max*_/σ^2^) becomes small, with faster convergence for larger *n* and smaller ν (see Fig. S1 for a broader range of *n*). Our rescaling relationships (5)–(7) assume equal‐sized clumps (Figs 1b,c insets), but we expect they will be robust to moderate perturbations of this idealised case, for example, unevenness in clump shape and size.

### Relating random clearing to contiguous clearing

We derived a further scaling result relating random‐clearing to contiguous‐clearing scenarios provided that σ ≫ 1:

(8) $$\begin{array}{c} S_{\mathrm{random}}\left(A_{\max},A,\nu ,\sigma^{2}\right)∼\;S_{\mathrm{contig}}\left(A,1-{\left(1-\nu \right)}^{\frac{A_{\max}}{A}},\sigma^{2}\right) \end{array}$$

This, our flagship theoretical result, is highly non‐trivial. To put it in words, if a fraction *A*/*A*_*max*_ of the landscape remains as habitat after a spatially random clearing event, this has a similar effect on species richness to a scenario of contiguous clearing leaving the same habitat area *A*, but on a more species‐rich landscape constructed with a larger speciation rate. This result can again be understood in terms of coalescence processes (Appendix 5).

Formula (8) gives a rescaling from random clearing to contiguous clearing. Formula (2) gives a rescaling between contiguous‐clearing scenarios with different sets of parameters. We can combine formulas (2) and (8) to get a rescaling between random‐clearing scenarios (Appendix 5):

(9) $$\begin{array}{c} S_{\mathrm{random}}\left(A_{\max},A,\nu ,\sigma^{2}\right)∼\frac{1}{k}S_{\mathrm{random}}\left(A_{\max},kA,1-{\left(1-\nu \right)}^{k},k\sigma^{2}\right) \end{array}$$

**Figure 5:**
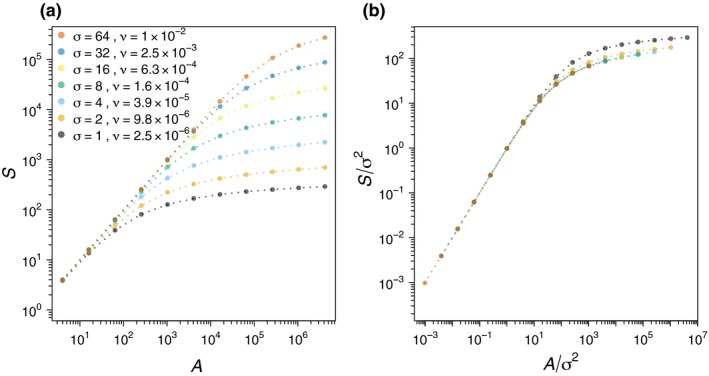
Panel a: SARs resulting from random clearing with parameters ν and σ related by $\nu =1-{\left(1-\nu_{0}\right)}^{k}$, where ν_0_ = 0.01, σ^2^ = 2^12^ *k* and *k* ∈ {2^−12^, 2^−10^, … 2^0^}. Panel b: The same SARs rescaled by 1/σ^2^ on both axes. These graphs demonstrate a different scaling collapse from that in Figs 2, 3, 4, because here the landscape extent is fixed (*A*_*max*_ = 2^22^) and only habitat *A* varies along the horizontal axis, and because the speciation rate ν now varies with dispersal standard deviation σ. As predicted by formula (9), a scaling collapse occurs in panel b and the collapse is better for large σ.

We verified numerically that this result holds true except for σ ≈ 1 (Fig. 5).

### Relating the results from all clearing patterns to the Preston function

The function $S_{\mathrm{contig}}\left(A,\nu ,\sigma^{2}\right)$ is already related to the two‐parameter Preston function by formula (1). The equivalent result for $S_{\mathrm{random}}\left(A_{\max},A,\nu ,\sigma^{2}\right)$ comes from a combination of formulas (1), (2) and (8):

(10) $$\begin{array}{c} S_{\mathrm{random}}\left(A_{\max},A,\nu ,\sigma^{2}\right)∼\;\sigma^{2}\Psi \left(\frac{A}{\sigma^{2}},1-{\left(1-\nu \right)}^{\frac{A_{\max}}{A}}\right) \end{array}$$

and is broadly valid for σ ≫ 1 (Fig. S2). This shows that the original four‐parameter random‐clearing function collapses on to the well‐studied two‐parameter Preston function and allows us to transfer knowledge about contiguous SARs to random‐clearing SARs. In the limits of large area and small speciation rate, our formulas for *S*_*contig*_ and *S*_*random*_ reduce to known SAR and endemics–area results for the log‐series distribution (He & Legendre 2002; Green & Ostling 2003; Appendix 6).

For clumped clearing there are two scenarios depending on the degree of dispersal limitation. If interpatch distance is large compared to the average species range *A*_*max*_/*n* ≫ σ^2^/ν, and σ^2^ ≫ 1, then we can combine formulas (1) and (5) to give

(11) $$\begin{array}{c} S_{\mathrm{clump}}\left(n,\;A_{\max},A,\nu ,\sigma^{2}\right)∼n\sigma^{2}\Psi \left(\frac{A}{n\sigma^{2}},\nu \right) \end{array}$$

In the opposite case *A*_*max*_/*n* ≪ σ^2^/ν, we can combine formulas (7) and (10) to give

(12) $$\begin{array}{c} S_{\mathrm{clump}}\left(n,A_{\max},A,\nu ,\sigma^{2}\right)∼\sigma^{2}\Psi \left(\frac{A}{\sigma^{2}},1-{\left(1-\nu \right)}^{\frac{A_{\max}}{A}}\right) \end{array}$$

Figure S3 summarises all the rescaling relationships derived in this study. The lower bound on species richness after habitat loss, and hence the upper bound on species loss, comes directly from the classic contiguous‐SAR approach by calculating $S_{\mathrm{contig}}\left(A,\nu ,\;\sigma^{2}\right)$. The upper bound on species richness, and hence the lower bound on species loss, comes from random‐clearing SARs by calculating $S_{\mathrm{random}}\left(A_{\max},A,\nu ,\sigma^{2}\right)$. Importantly, both can now be expressed analytically in terms of the well‐studied Preston function.

### Local‐scale findings and case study: Barro Colorado Island, Panama

**Figure 6:**
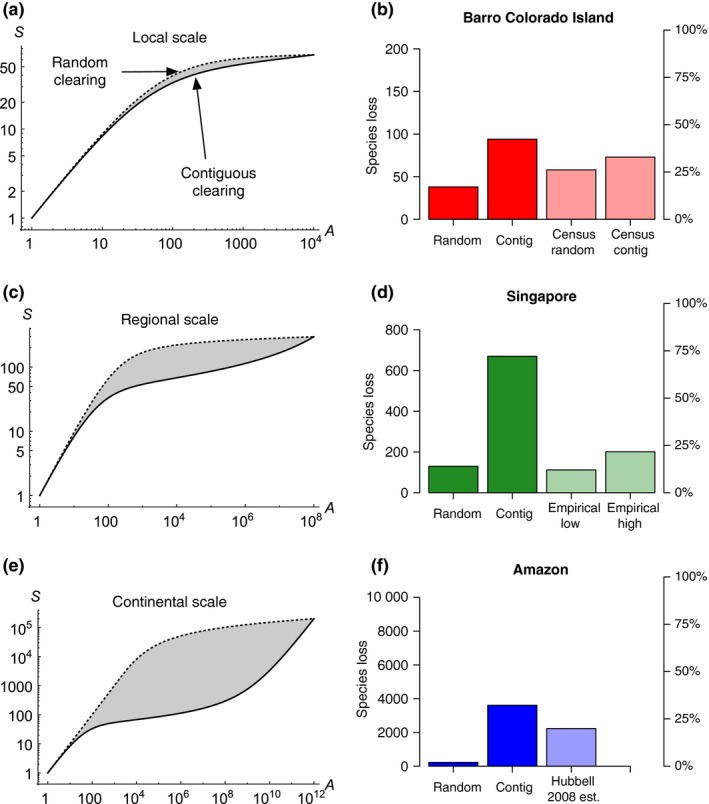
Results from our random‐clearing and contiguous‐clearing SAR formulas with realistic parameter values (see formula (8), and formula (S1) in Appendix 1). Panels a, c and e show illustrative random‐clearing SARs (dashed curves) and contiguous‐clearing SARs (solid curves) with landscape area *A*_*max*_ = 10^4^, 10^8^ and 10^12^, respectively, and σ = 8 and ν = 10^−8^. Panels b, d and f show estimated tree species loss in our case studies at the local scale (hypothetical forest loss of 90% in Barro Colorado Island (BCI) 50 ha plot, Panama), regional scale (observed forest loss in Singapore, 1819–present) and continental scale (observed forest loss in the Brazilian Amazon since pre‐1970), with estimates from our formulas (dark coloured bars) compared to estimates from other methods (light coloured bars; see main text for parameterisations and other details of the case studies).

We observed that on local scales, random and contiguous clearing give qualitatively similar SARs (Fig. 6a); thus fragmented SARs will be similar too, because random and contiguous clearing are the bounding cases. Despite the qualitative similarity of the SARs, the quantitative differences in species loss estimates between clearing scenarios can be fairly large (e.g. by a factor of ≈ 2.5 for 90% forest loss in the BCI plot; Fig. 6b). When we compared the predictions from the formulas to the results of simulated clearing on the spatial census data, we found that the simulated results were within the bounds from the formulas, but showed less variation between the random and contiguous scenarios (Figs 6b; S4, Appendix 8). This discrepancy is expected, given the known limitations of current spatial neutral models at small scales, that is, their failure to accurately reproduce small‐scale beta diversity patterns (Condit *et al*. 2002) and species abundance distributions (O'Dwyer & Cornell 2017).

### Regional‐scale findings and case study: Singapore

When clearing occurs at a regional scale, qualitative differences between the random and contiguous scenarios emerge (Fig. 6c), and the quantitative differences become larger. In our application to Singapore, estimated species loss in the contiguous‐clearing scenario is five times that in the random‐clearing scenario, and empirical tree species loss is closer to the random‐clearing lower bound (Fig. 6d).

### Continental‐scale findings and case study: the Brazilian Amazon

At continental scales, where the third phase of the SAR dominates, major qualitative differences arise in the SAR across fragmentation types: while the contiguous model predicts that the proportion of species lost equals the proportion of area lost in the initial stages of habitat destruction, the random‐clearing model predicts very little species loss until the vast majority of habitat has been destroyed (Fig. 6e). The stark differences between the predictions of the random and contiguous models leave a huge envelope of possible intermediate outcomes for fragmented‐clearing scenarios (Fig. 6f).

## Discussion

How many species are lost when an area of grassland goes under the plough or a forest falls by the axe? Ecologists have pondered the species–area question for almost a century (Arrhenius 1921), but have only recently come to grapple with the spatial nature of the problem, with the help of advanced computer simulations and mathematics (Durrett & Levin 1996; Rosindell & Cornell 2007; O'Dwyer & Green 2010; Grilli *et al*. 2012). Here, we have compartmentalised the problem by first designating the ‘Preston function’ to be the well‐studied triphasic contiguous SAR that emerges from a spatial neutral model, and then deriving new formulas that relate this existing work to a range of new fragmented SAR scenarios (Fig. S3). Our results are derived from neutral models, but their applicability extends to any situation where the spatial patterns of species distributions are statistically similar to those of neutral species – the actual mechanisms generating the patterns need not be neutral.

The spatial pattern of habitat loss can have a major influence on species loss, and traditional contiguous SARs may overestimate this loss (Kinzig & Harte 2000), but most studies on fragmented SARs have been restricted to phenomenological approaches or numerical simulations. Although useful, simulations come with computational limitations that restrict the spatial scale and resolution at which questions can be asked and the generality of conclusions drawn. While our local‐scale case studies (Fig. 6a,b) could easily be investigated numerically, our regional‐scale case studies (Fig. 6c,d) would be more difficult to simulate at the resolution of individuals, and our continental‐scale case studies (Fig. 6e,f) would be impossible with present technology: we estimate that even efficient neutral coalescence simulations of our Amazon case study would take millennia on a modern desktop machine, whereas our analytical formulas can be evaluated in milliseconds. Our method allows for rapid estimation of upper and lower bounds on species loss from fragmented habitat clearing, with the upper bound given by a contiguous SAR and the lower bound given by a random‐clearing SAR (see Appendix 7 for a technical discussion).

Our case studies reveal a strong scale‐dependence to the fragmented SAR problem: while the pattern of clearing makes only a moderate difference to species loss on local scales (Fig. 6a,b), the effect on regional and, especially, continental scales can be enormous (Fig. 6c,f). The scale of observation also affects whether empirical species loss from fragmented clearing is closer to the theoretical lower or upper bound. In the idealised case where species are distributed randomly at the scale of observation, species loss is predicted exactly from the lower bound, corresponding to the random SAR, under any spatial pattern of habitat destruction (He & Hubbell 2011). In more realistic cases, where species distributions have some characteristic scale, the random SAR formula will continue to work well providing that this characteristic scale is substantially greater than the scale of clumping in the habitat destruction spatial pattern (formula (7)). This explains why empirical tree species loss in Singapore is close to the theoretical random‐clearing lower bound (Fig. 6c,d): the average tree species’ range is much larger than the distance between forest clumps in Singapore.

At continental scales, the range of possible outcomes from fragmented clearing becomes enormous (Fig. 6e) and, lacking empirical data on species loss, we cannot be certain whether our contiguous‐clearing upper bound or the random‐clearing lower bound is more accurate. This is because the condition for the clumped‐to‐random scaling collapse (formula (7)) may fail at large scales: the typical species ranges may cover just one or a few clumps. It is also at continental scales that the power and utility of our methods become fully apparent. In the Amazon, biologists lack certainty about the number of extant tree species, let alone the extinct ones. In such cases, if we want to estimate tree diversity and extinction, we must leverage theoretical models, despite their inevitable caveats (Hubbell *et al*. 2008).

Two important general lessons emerge from the application of our new methods to the three case studies at different scales. First, the relationship between fragmentation and immediate species loss is fundamentally scale dependent: fragmentation has only moderate effects independent of area at small scales (Fig. 6a), but enormous effects at large scales (Fig. 6e). Second, the 16‐fold difference between our lower bound random‐clearing and upper bound contiguous‐clearing predictions at the Amazon scale (Fig. 6f) illustrates starkly that we can have no hope of even approximately characterising continental‐scale species loss unless we come to grips with the mathematics of fragmented SARs.

We focused on immediate species loss from habitat destruction here, but there is also a need to study long‐term loss (e.g. Claudino *et al*. 2015). Immediate species loss may be more relevant for communities of long‐lived sedentary organisms, such as trees, or small‐bodied organisms that can persist in tiny patches of habitat. But long‐term loss is important for communities of organisms that are mobile or turn over relatively quickly, for example, birds and mammals. Critically, the effects of fragmentation on long‐term loss can be opposite to those on immediate loss, with diversity negatively related to fragmentation (Hanski *et al*. 2013). Long‐term loss is a more complex technical problem, because it necessarily includes immediate loss as one component, but also involves a richer cocktail of biological processes, including edge effects and population structure. Our methods can be extended to study cases where long‐term relaxation occurs via neutral drift, but a comprehensive evaluation of long‐term loss will likely involve a broader suite of modelling techniques.

In summary, we have introduced a suite of formulas that allow for analytical, mechanistic estimation of lower and upper bounds on immediate species loss at arbitrary spatial scales. The resulting insights about fragmented SARs help provide a foundation for future work on this topic. Our formulas predict, consistent with other models and with intuition, that immediate species loss is lower under random clearing than under contiguous clearing, and in between for other cases of fragmented clearing. We have shown that true species loss will, all else being equal, be closer to the lower bound (random clearing) than the upper bound (contiguous clearing) for (1) well‐dispersed taxa, (2) highly fragmented landscapes and (3) small spatial scales. Our rescaling formulas advance research on fragmented SARs by providing an analytical way forward on a topic where analytical solutions have been scarce.

## Authorship

RAC and JR designed the study. RAC, JR and FL performed the analyses. YSY, WWS and RC provided and collated the data. RAC and JR wrote the paper. All authors revised the paper.

## References

[ele12943-bib-0001] Arrhenius, O. (1921). Species and area. J. Ecol., 9, 95–99.

[ele12943-bib-0002] Campos, P.R.A. , Neto, E.D.C. , Oliveira, V.M. & Gomes, M.A.F. (2012). Neutral communities in fragmented landscapes. Oikos, 121, 1737–1748.

[ele12943-bib-0003] Campos, P.R.A. , Rosas, A. , de Oliveira, V.M. & Gomes, M.A.F. (2013). Effect of landscape structure on species diversity. PLoS ONE, 8, e66495. 23840490 10.1371/journal.pone.0066495 PMC3686687

[ele12943-bib-0004] Chisholm, R.A. , Giam, X. , Sadanandan, K.R. , Fung, T. & Rheindt, F.E. (2016). A robust non‐parametric method for quantifying undetected extinctions. Conserv. Biol., 30, 610–617. 27153528 10.1111/cobi.12640

[ele12943-bib-0005] Claudino, E.S. , Gomes, M.A.F. & Campos, P.R.A. (2015). Extinction debt and the role of static and dynamical fragmentation on biodiversity. Ecol. Complex., 21, 150–155.

[ele12943-bib-0006] Condit, R. , Pitman, N. , Leigh, E.G. , Chave, J. , Terborgh, J. , Foster, R.B. *et al* (2002). Beta‐diversity in tropical forest trees. Science, 295, 666–669. 11809969 10.1126/science.1066854

[ele12943-bib-0007] Corlett, R.T. (1992). The ecological transformation of Singapore, 1819‐1990. J. Biogeogr., 19, 411–420.

[ele12943-bib-0008] Durrett, R. & Levin, S. (1996). Spatial models for species‐area curves. J. Theor. Biol., 179, 119–127.

[ele12943-bib-0009] Ewers, R.M. & Laurance, W.F. (2006). Scale‐dependent patterns of deforestation in the Brazilian Amazon. Environ. Conserv., 33, 203–211.

[ele12943-bib-0010] Gardner, R.H. , Milne, B.T. , Turner, M.G. & O'Neill, R.V. (1987). Neutral models for the analysis of broad‐scale landscape pattern. Landsc. Ecol., 1, 19–28.

[ele12943-bib-0011] Green, J.L. & Ostling, A. (2003). Endemics‐area relationships: the influence of species dominance and spatial aggregation. Ecology, 84, 3090–3097.

[ele12943-bib-0012] Grilli, J. , Azaele, S. , Banavar, J. & Maritan, A. (2012). Absence of detailed balance in ecology. EPL, 100, 38002.

[ele12943-bib-0013] Hanski, I. , Zurita, G.A. , Bellocq, M.I. & Rybicki, J. (2013). Species–fragmented area relationship. Proc. Natl Acad. Sci., 110, 12715–12720. 23858440 10.1073/pnas.1311491110 PMC3732936

[ele12943-bib-0014] Hargrove, W.W. , Hoffman, F.M. & Schwartz, P.M. (2002). A fractal landscape realizer for generating synthetic maps. Conservation Ecology, 6(1), 2 [online] URL: http://www.consecol.org/vol6/iss1/art2/

[ele12943-bib-0015] Harte, J. , Smith, A.B. & Storch, D. (2009). Biodiversity scales from plots to biomes with a universal species–area curve. Ecol. Lett., 12, 789–797. 19486123 10.1111/j.1461-0248.2009.01328.x

[ele12943-bib-0016] He, F. & Hubbell, S.P. (2011). Species–area relationships always overestimate extinction rates from habitat loss. Nature, 473, 368–371. 21593870 10.1038/nature09985

[ele12943-bib-0017] He, F. & Hubbell, S.P. (2013). Estimating extinction from species‐area relationships: why the numbers do not add up. Ecology, 94, 1905–1912. 24279261 10.1890/12-1795.1

[ele12943-bib-0018] He, F.L. & Legendre, P. (2002). Species diversity patterns derived from species‐area models. Ecology, 83, 1185–1198.

[ele12943-bib-0019] Hubbell, S.P. (2001). The Unified Neutral Theory of Biodiversity and Biogeography. Princeton University Press, Princeton, NJ.

[ele12943-bib-0020] Hubbell, S.P. , Condit, R. & Foster, R.B. (2005). Barro Colorado Forest Census Plot Data, URL http://ctfs.si/edu/datasets/bci.

[ele12943-bib-0021] Hubbell, S.P. , He, F.L. , Condit, R. , Borda‐de‐Agua, L. , Kellner, J. & ter Steege, H. (2008). How many tree species are there in the Amazon and how many of them will go extinct? Proc. Natl Acad. Sci. USA, 105, 11498–11504. 18695228 10.1073/pnas.0801915105 PMC2556410

[ele12943-bib-0022] Keil, P. , Storch, D. & Jetz, W. (2015). On the decline of biodiversity due to area loss. Nat. Commun., 6, 8837 10.1038/ncomms9837. 26575347 PMC4660053

[ele12943-bib-0023] Kinzig, A.P. & Harte, J. (2000). Implications of endemics‐area relationships for estimates of species extinctions. Ecology, 81, 3305–3311.

[ele12943-bib-0024] Lewis, O.T. (2006). Climate change, species‐area curves and the extinction crisis. Philosophical Transactions of the Royal Society B: Biological Sciences, 361, 163–171. 10.1098/rstb.2005.1712 PMC1831839 16553315

[ele12943-bib-0025] Muller‐Landau, H.C. , Wright, S.J. , Calderon, O. , Condit, R. & Hubbell, S.P. (2008). Interspecific variation in primary seed dispersal in a tropical forest. J. Ecol., 96, 653–667.

[ele12943-bib-0026] O'Dwyer, J.P. & Chisholm, R.A. (2013). Neutral theory and beyond In: Encyclopedia of Biodiversity, 2nd edn (ed. Levin S.A.). Academic Press, Waltham, MA, 51pp.

[ele12943-bib-0027] O'Dwyer, J.P. & Cornell, S. (2017). Cross‐scale ecological theory sheds light on the maintenance of biodiversity. https://arxiv.org/abs/1705.07856.

[ele12943-bib-0028] O'Dwyer, J.P. & Green, J.L. (2010). Field theory for biogeography: a spatially explicit model for predicting patterns of biodiversity. Ecol. Lett., 13, 87–95. 19909313 10.1111/j.1461-0248.2009.01404.x PMC2810436

[ele12943-bib-0029] Pereira, H.M. & Daily, G.C. (2006). Modeling biodiversity dynamics in countryside landscapes. Ecology, 87, 1877–1885. 16937624 10.1890/0012-9658(2006)87[1877:mbdicl]2.0.co;2

[ele12943-bib-0030] Pereira, H.M. , Borda‐de‐Agua, L. & Martins, I.S. (2012). Geometry and scale in species‐area relationships. Nature, 482, E3–E4. 22358846 10.1038/nature10857

[ele12943-bib-0031] Plotkin, J.B. , Potts, M.D. , Leslie, N. , Manokaran, N. , LaFrankie, J. & Ashton, P.S. (2000). Species‐area curves, spatial aggregation, and habitat specialization in tropical forests. J. Theor. Biol., 207, 81–99. 11027481 10.1006/jtbi.2000.2158

[ele12943-bib-0032] Preston, F.W. (1960). Time and space and the variation of species. Ecology, 41, 611–627.

[ele12943-bib-0033] Rosindell, J. & Cornell, S.J. (2007). Species‐area relationships from a spatially explicit neutral model in an infinite landscape. Ecol. Lett., 10, 586–595. 17542937 10.1111/j.1461-0248.2007.01050.x

[ele12943-bib-0034] Rosindell, J. , Wong, Y. & Etienne, R.S. (2008). A coalescence approach to spatial neutral ecology. Ecological Informatics, 3, 259–271.

[ele12943-bib-0035] Storch, D. , Keil, P. & Jetz, W. (2012). Universal species‐area and endemics‐area relationships at continental scales. Nature, 488, 78–81. 22722856 10.1038/nature11226

[ele12943-bib-0036] Sugihara, G. & May, R.M. (1990). Applications of fractals in ecology. Trends Ecol. Evol., 5, 79–86. 21232328 10.1016/0169-5347(90)90235-6

[ele12943-bib-0037] With, K.A. (1997). The application of neutral landscape models in conservation biology. Conserv. Biol., 11, 1069–1080.

[ele12943-bib-0038] Zillio, T. & He, F. (2010). Modeling spatial aggregation of finite populations. Ecology, 91, 3698–3706. 21302840 10.1890/09-2233.1

