# Supplementary material for: Species–area relationships and biodiversity loss in fragmented landscapes
Source: Ecol Lett. 2018 Mar 30;21(6):804–13. doi: 10.1111/ele.12943 (PMC6849768; doi:10.1111/ele.12943)
Supplement: Supplementary file 1 [file ELE-21-804-s001.docx]

**Appendices to “Species–area Relationships and Biodiversity Loss**

**in Fragmented Landscapes”**

Ryan A. Chisholm, Felix Lim, Yi Shuen Yeoh, Wei Wei Seah, Richard Condit, and James Rosindell

**Appendix 1: Analytical solution to the Preston function**

To generate the results for our case studies (Fig. 6), we used a published analytical result for the Preston function that is derived via an approximation scheme that applies different linearizations in different regions of parameter space (O'Dwyer & Cornell 2017):

$$\begin{aligned} \Psi\left( A^{*},\nu\right)\approx\nu_{eff}A^{*}+\frac{2\pi\sqrt{\frac{A^{*}}{\pi}}\left( 1-\nu_{eff} \right)I_{1}\left( \sqrt{\frac{A^{*}}{\pi}} \right)}{\frac{1}{\sqrt{\nu_{eff}}}I_{1}\left( \sqrt{\frac{A^{*}}{\pi}} \right)\frac{K_{0}\left( \sqrt{\frac{\nu_{eff}A^{*}}{\pi}} \right)}{K_{1}\left( \sqrt{\frac{\nu_{eff}A^{*}}{\pi}} \right)}+I_{0}\left( \sqrt{\frac{A^{*}}{\pi}} \right)} \end{aligned}$$

(S1)

Here $A^{*}=A/\sigma^{2}$ is area measured in units corresponding to the variance of dispersal distance, $\nu_{eff}=\nu\log\left( 1/\nu\right)/(1-\nu)$, $\nu$ is the per-capita speciation rate, and $I_{i}(z)$ and $K_{i}(z)$ are the modified Bessel functions of the first and second kinds.

**Appendix 2: parameterizing the model**

In this appendix, we describe how to parameterize our model. There are three parameters that must be estimated: the density of individuals per unit area $\rho$, the standard deviation of dispersal distance $\sigma$, and the speciation rate $\nu$. The parameters $\sigma$ and $\nu$ appear directly in Eq. (S1). The parameter $\rho$ sets the scale at which all area- and distance-based variables are measured, i.e., in all of our formulas the units of area are rescaled by $\rho$ such that there is one individual per unit area.

Estimating $\rho$ is straightforward in principle: one must just count the number of individuals per unit area in the system of interest. If fieldwork is impractical, one can estimate $\rho$ from published sources for similar systems. In our applications to tropical forests, we were able to draw on published estimates of $\rho$ from plot census data. For other taxa in other systems, estimates of $\rho$ can be taken from, e.g., Chisholm et al. (2016, Appendix S3) and references therein.

Estimating the dispersal scale $\sigma$ is more challenging. There are two broad sets of approaches. The first assumes that the dynamics of the system are close to neutral and that therefore the $\sigma$ parameter can be interpreted literally as the standard deviation of dispersal distance and can be measured directly in the field. Methods for estimating dispersal parameters in the field include seed traps for trees (Muller-Landau *et al.* 2008) and radio telemetry for vertebrates (Marvin *et al.* 2016). However, these methods are likely to be prohibitively costly in most situations where the data are not pre-existing.

The second broad set of approaches for estimating $\sigma$ assumes that the statistical patterns of species distributions are similar to neutral species, but allows that the dynamics of the system itself may not be neutral and that $\sigma$ may thus be an effective parameter that is affected not only by dispersal but also environmental heterogeneity and other factors. In this case, $\sigma$ can be estimated from the spatial distribution of individuals in the landscape. Condit *et al.* (2002) did this by taking a mathematical expression for the probability that two individuals at a distance $r$ in a neutral model are conspecific, and fitting the two parameters $\sigma$ and $\nu$ to tree census data from 34 forest plots separated by distances of up to 50 km in the vicinity of the Panama Canal (see also Chave & Leigh 2002; O'Dwyer & Green 2010). We used the estimate of $\sigma$ from Condit *et al.* in the main text and in general we recommend this approach to estimating $\sigma$, where the required census data are available, simply because it does not assume that the actual dynamics of the system are neutral and is thus more likely to generate robust results. As a fallback approach, if the data required to estimate $\sigma$ are not available, we recommend exploring the robustness of the species loss predictions by using a range of plausible $\sigma$ values. In many cases, the values of $\sigma$ may be transferable across systems, e.g., in our study we assumed that the average dispersal distances of tropical trees do not differ greatly across the world.

To estimate the speciation rate $\nu$, which controls large-scale diversity, the approach of Condit et al. (2002) described above is also effective. A limitation here is that the values of $\nu$, unlike values of $\sigma$, do not transfer readily across systems. For example, in our study we could not assume that $\nu$ was the same across the tropics because tree species richness exhibits large regional variation. A reasonable approach, and the one we used, is instead to first estimate $\rho$ and $\sigma$ as described above, leaving $\nu$ as the only free parameter, and then to estimate $\nu$ from a single constraint, typically the known value of species richness $(S)$ at a particular scale ($A$). To do this, one simply plugs in $S$, $A$, $\rho$ and $\sigma$ into Eq. (S1) and estimates $\nu$ using numerical root-finding methods (e.g., see our code in Appendix 3).

We emphasize that our model predictions of species loss with habitat loss will be reliable only if (i) the study system is truly neutral; or (ii) the study system is not neutral but species distributions nevertheless approximately resemble those of neutral species. In the latter case, the parameters (especially $\sigma$) must be treated as effective parameters and estimated indirectly from the actual species’ spatial distributions rather than directly from field data. If these requirements are not met, e.g., if there is no evidence that species’ spatial distributions are fit well by a neutral model, then the predictions of our model come with strong caveats. For example, species that are locally common but confined to small patches of sparsely scattered habitat have distinctly non-neutral spatial distributions; the reliability of our methods applied to such situations is unknown, pending further research.

**Appendix 3: R code**

This R code implements our contiguous-clearing and random-clearing SARs to produce upper and lower bounds on species loss for our three case studies. The code can be adapted for other applications by changing the values of the parameters rho, sigma, A_max, S_max and A in the call to the function estimate_species_loss().

rm(list=ls())

Preston <- function(A_on_sigma_sq,nu)

{

a = sqrt(A_on_sigma_sq/pi)

nu_eff = nu*log(1/nu)/(1-nu)

num = 2*pi*a*(1-nu_eff)*besselI(a,1,expon.scaled=T)

denom = (1/sqrt(nu_eff))*besselI(a,1,expon.scaled=T)*besselK(sqrt(nu_eff)*a,0,expon.scaled=T)/besselK(sqrt(nu_eff)*a,1,expon.scaled=T)+besselI(a,0,expon.scaled=T)

return(nu_eff*A_on_sigma_sq+num/denom)

}

S_contig <- function(A,nu,sigma_sq)

{

sigma_sq*Preston(A/sigma_sq,nu)

}

S_random <- function(A_max,A,nu,sigma_sq)

{

sigma_sq*Preston(A/sigma_sq,1-(1-nu)^(A_max/A))

}

# A_max, A and sigma^2 must have the same units

estimate_species_loss_scalar <- function(A_max,S_max,rho,sigma,A)

{

epsilon_lo = .Machine$double.xmin

epsilon_hi = .Machine$double.neg.eps

temp = uniroot(function(log_nu_est) S_contig(A_max*rho,exp(log_nu_est),sigma^2*rho)-S_max,lower=log(epsilon_lo),upper=log(1-epsilon_hi))

nu = exp(temp$root)

S_est_contig = S_contig(A*rho,nu,sigma^2*rho)

if ( !is.finite(S_est_contig) ) warning("Could not estimate species loss from contiguous clearing: parameters exceed numerical limits\n")

S_est_random = S_random(A_max*rho,A*rho,nu,sigma^2*rho)

if ( !is.finite(S_est_random) ) warning("Could not estimate species loss from random clearing: parameters exceed numerical limits\n")

r_val = list()

r_val["S_original"] = S_max

r_val["S_final lower bound (contiguous clearing)"] = S_est_contig

r_val["S_final upper bound (random clearing)"] = S_est_random

r_val["S_loss lower bound (random clearing)"] = S_max-S_est_random

r_val["S_loss upper bound (contiguous clearing)"] = S_max-S_est_contig

r_val

}

estimate_species_loss <- Vectorize(estimate_species_loss_scalar)

# tropical forest trees

rho = 42000 # km^-2

sigma = 0.0402 # km

# BCI

A_max_BCI = 0.5 # km^2

S_max_BCI = 222

A_BCI = 0.05 # km^2

# Singapore

A_max_Singapore = 540 # km^2

S_max_Singapore = 840+89

A_Singapore = 23 # km^2

# Amazon

A_max_Amazon = 4652400 # km^2

S_max_Amazon = 11210

A_Amazon = 0.633*A_max_Amazon # km^2

A_maxs = c(A_max_BCI,A_max_Singapore,A_max_Amazon)

names(A_maxs) = c('BCI','Singapore','Amazon')

S_maxs = c(S_max_BCI,S_max_Singapore,S_max_Amazon)

As = c(A_BCI,A_Singapore,A_Amazon)

print(estimate_species_loss(A_maxs,S_maxs,rho,sigma,As))

**Appendix 4: Spatial scaling results for arbitrary clearing patterns**

Here we investigate whether the rescaling formula for the contiguous SAR (formula (2)) originally discovered by Rosindell & Cornell (2007) works for arbitrary clearing patterns. Specifically, we test whether increasing all spatial parameters (total landscape area $A_{max}$, habitat area $A$, and dispersal scale $\sigma^{2}$) by a factor $k$ leads to an increase of species richness also by a factor of $k$, when the dispersal scale $\sigma$ is not too small.

We consider five distinct clearing patterns: clumped clearing (see main text; Fig. 1B–C insets) with baseline $A_{max}=16,384$, $A=512, n=16$, and four other clearing patterns also with baseline $A_{max}=16,384$ (but each with a different $A$; Fig. 4). For the community landscapes, we conducted spatially explicit neutral coalescence simulations (Rosindell *et al.* 2008) with $\nu=0.01$, and $\sigma\in\{1,2,4,8,16,32\}$ and 10 repeats for each value of $\sigma$.

For the baseline case ($A_{max}=16,384$), we applied each clearing map to a landscape with $\sigma_{0}=1$. For each successive value of $\sigma$, we stretched each clearing map by a factor $\sqrt{k}=\sigma/\sigma_{0}$ in each dimension and generated new community landscapes with matching area. When all the simulations had been run, we found, as predicted, that, for sufficiently large $\sigma$, the value of $S/\sigma^{2}$ (where $S$ is species richness) was roughly constant across simulations for a particular clearing map, confirming that the rescaling formula originally derived for contiguous SARs (formula (2)) also applies to arbitrary fragmented SARs.

To understand this result intuitively, imagine stretching all the spatial dimensions of a simulated community by a factor of $\sqrt{k}$, where $k$ is an arbitrary scaling parameter. This means area parameters, and the variance of the dispersal distance, all increase by a factor of $k$ (formulas (3) and (4)). Provided that the scale of dispersal is significantly larger than an individual ($\sigma^{2}\gg1$) so that species never form impenetrable patches of monoculture, each species is stretched out over a larger area, but otherwise remains governed by the same fundamental abundance dynamics as before. The stretching out of each species’ range without changing its abundance leaves space that will be filled with more species governed by the same rules. With area, and hence total number of individuals, expanded by a factor of $k$, this will in turn yield $k$ times the number of species compared to when there was no stretching of space (see the right-hand sides of formulas (3) and (4)).

**Appendix 5: Derivation of scaling results for random clearing**

Here we derive formulas (8) and (9) from the main text, i.e., the formulas giving the random-to-contiguous and random-to-random rescalings. The derivation of formula (8) relies on an understanding of the coalescence processes that can be used to simulate the underlying neutral model. To calculate species richness of remaining habitat using a coalescence process we trace the genealogies of individuals in the remaining habitat cells back through time until they converge on a common ancestor (Rosindell *et al.* 2008). Each time step of the coalescence process involves a random jump in space drawn from the dispersal kernel with variance $\sigma^{2}$. The individuals in the remaining habitat cells are represented as tips on a coalescent tree, and the species richness of remaining habitat is determined by the structure of the tree and the per-capita per-generation speciation rate parameter $\nu$.

In the contiguous-clearing case, the structure of the coalescent tree, and the resulting species richness, can be calculated using established methods (Rosindell *et al.* 2008). Our new insight here is that the random-clearing case is similar to the contiguous-clearing case after an appropriate rescaling. In the random clearing case, the lineages $P$ we are tracing back through time are less dense in space by a factor $A/A_{max}$. Consequently, there are fewer lineages available to coalesce with each other and the probability of a particular lineage in $P$ coalescing with any other in a single timestep is lower than the contiguous case by a factor $A/A_{max}$. This implies that the total waiting time to coalescence for each lineage under random clearing will be greater by the same factor $A/A_{max}$. The net effect is simply to stretch out the coalescence tree and all its branches by $A/A_{max}$ in the time dimension.

Now compare the contiguous- and random-clearing cases and consider how the diversity in the remaining habitat in each case is structured by imposing a speciation process on the coalescent tree. Let $T$ be the expected frequency distribution of branch lengths in a coalescent tree from a contiguous landscape. The probability of at least one speciation event along a branch of length $\tau\in T$ is $1-\left( 1-\nu\right)^{\tau}$. In the random-clearing case with the same total number of individuals (same number of tips), all branch lengths are stretched out by a factor $A_{max}/A$ but the tree is otherwise equivalent, so the branch lengths follow a frequency distribution $TA_{max}/A$. And thus the distribution of speciation probabilities along branches in the random-clearing case comes from $1-\left( 1-\nu\right)^{\tau A_{max}/A}$, where $\tau\in T$. But then we have

$$1-\left( 1-\nu\right)^{\tau\frac{A_{max}}{A}}=1-\left( 1-\nu^{'} \right)^{\tau}$$

where $\nu^{'}=1-\left( 1-\nu\right)^{\frac{A_{max}}{A}}$, which shows that the random-clearing case with speciation rate $\nu$ is equivalent to the contiguous-clearing case with a different speciation rate $\nu^{'}$. This insight yields formula (8):

$S_{random}\left( A_{max},A,\nu,\sigma^{2} \right)\sim S_{contig}\left( A,1-\left( 1-\nu\right)^{\frac{A_{max}}{A}},\sigma^{2} \right)$

A side note: the arguments applied to random clearing here do not necessarily work for arbitrary clearing patterns (e.g., clumped clearing), where the distribution of branch lengths $T$ may be idiosyncratic, with a dependence on the shape of the dispersal kernel and the degree of spatial autocorrelation present at different scales in the distribution of remaining habitat.

Now we show how the random-to-contiguous rescaling result (formula (8)) leads to a random-to-random rescaling result (formula (9)). First use formula (2) from the main text, which gives a rescaling between contiguous-clearing scenarios with different sets of parameters:

$$S_{contig}\left( A,1-\left( 1-\nu\right)^{\frac{A_{max}}{A}},\sigma^{2} \right)\sim\frac{1}{k}S_{contig}\left( kA,1-\left( 1-\nu\right)^{\frac{A_{max}}{A}},k\sigma^{2} \right)$$

for any arbitrary scaling parameter $k$. Manipulating the right-hand side gives

$$S_{contig}\left( A,1-\left( 1-\nu\right)^{\frac{A_{max}}{A}},\sigma^{2} \right)\sim\frac{1}{k}S_{contig}\left( kA,1-\left( \left( 1-\nu\right)^{k} \right)^{\frac{A_{max}}{kA}},k\sigma^{2} \right)$$

Now applying formula (8) on both sides gives

$S_{random}\left( A_{max},A,\nu,\sigma^{2} \right)\sim\frac{1}{k}S_{random}(A_{max},kA,1-\left( 1-\nu\right)^{k},k\sigma^{2})$

As expected, this rescaling gives accurate results except for values of $\sigma$ approaching 1, the scale of the individual (see Fig. 5 of the main text).

**Appendix 6: Comparison to known log-series results**

In this appendix, we show that known results concerning the SAR and the endemics–area relationship for the log-series species abundance distribution (Fisher *et al.* 1943; He & Legendre 2002; Green & Ostling 2003) emerge as special cases of our formulas in the limit of large landscape area.

*Contiguous SAR*

Let us start with our SAR formula for contiguous areas (formula (1); O'Dwyer & Cornell 2017) and compute the species richness of an entire landscape of area $A_{max}$:

$$\begin{aligned} S_{contig}\left( A_{max},\nu,\sigma^{2} \right)\sim\sigma^{2}\Psi\left( \frac{A_{max}}{\sigma^{2}},\nu\right) \end{aligned}$$

From formula (S1) for the Preston function $\Psi$, we find the large-area ($A_{max}\to\infty$) asymptotic solution

$$S_{contig}\left( A_{max},\nu,\sigma^{2} \right)\sim\frac{A_{max}\nu}{1-\nu}\log\left( \frac{1}{\nu} \right)$$

(S2)

This is the known result for species richness from a log-series distribution (Fisher *et al.* 1943):

$$S=-\alpha log(1-x)$$

where $\alpha=A_{max}\nu/(1-\nu)$ (from the standard formula for the fundamental biodiversity number in neutral theory) and $x=1-\nu$.

*Random SAR*

Now imagine that we are clearing randomly within the landscape of area $A_{max}$, and consider our random SAR formula (formula (10)):

$$\begin{aligned} S_{random}\left( A_{max},A,\nu,\sigma^{2} \right)\sim\sigma^{2}\Psi\left( \frac{A}{\sigma^{2}},1-\left( 1-\nu\right)^{\frac{A_{max}}{A}} \right) \end{aligned}$$

Asymptotically, for large area ($A\to\infty$, which also implies $A_{max}\to\infty$) we have

$$S_{random}\left( A_{max},A,\nu,\sigma^{2} \right)\sim\frac{1-\left( 1-\nu\right)^{\frac{A_{max}}{A}}\log\left( \frac{1}{1-\left( 1-\nu\right)^{\frac{A_{max}}{A}}} \right)}{1-\left( 1-\left( 1-\nu\right)^{\frac{A_{max}}{A}} \right)}A$$

For small values of the speciation rate ($\nu\to0$), this can be approximated as follows:

$$S_{random}\left( A_{max},A,\nu,\sigma^{2} \right)\approx\frac{\frac{A_{max}}{A}\nu\log\left( \frac{A}{A_{max}\nu} \right)}{1-\frac{A_{max}}{A}\nu}A\approx A_{max}\nu\log\left( \frac{A}{A_{max}\nu} \right)$$

(S3)

We can compare this to the known SAR result from the log-series (He & Legendre 2002) with the same definitions of $\alpha$ and $x$ as above and again assuming the speciation rate is small ($\nu\to0$):

$$S=\alpha\log\left( 1+\frac{x}{1-x}\frac{A}{A_{max}} \right)=\frac{A_{max}\nu}{1-\nu}\log\left( 1+\frac{1-\nu}{\nu}\frac{A}{A_{max}} \right)\approx A_{max}\nu\log\left( \frac{A}{A_{max}\nu} \right)$$

And we thus see that our formula is equivalent to the known result in the limits of large area and small speciation rate.

*Random endemics–area relationship*

Now consider the endemics–area relationship for random clearing:

$$S_{loss,random}\left( A_{max},A,\nu,\sigma^{2} \right)=E_{random}\left( A_{max},A,\nu,\sigma^{2} \right)=S_{contig}\left( A_{max},\nu,\sigma^{2} \right)-S_{random}\left( A_{max},A_{max}-A,\nu,\sigma^{2} \right)\approx\frac{\nu\log\left( \frac{1}{\nu} \right)}{1-\nu}A_{max}-\frac{A_{max}\nu}{1-\nu}\log\left( \frac{A_{max}-A}{A_{max}\nu} \right)=-\frac{A_{max}\nu}{1-\nu}\log\left( 1-\frac{A}{A_{max}} \right)$$

(S4)

where again the approximation is valid for small $\nu$. This can be compared to the known log-series endemics–area result (Green & Ostling 2003):

$$E\left( A \right)=-\alpha\log\left( 1-x\frac{A}{A_{max}} \right)=-\frac{A_{max}\nu}{1-\nu}\log\left( 1-\left( 1-\nu\right)\frac{A}{A_{max}} \right)\approx-\frac{A_{max}\nu}{1-\nu}\log\left( 1-\frac{A}{A_{max}} \right)$$

where again the approximation holds for small $\nu$, giving the same result as (S4).

**Appendix 7: Technical discussion of results**

Here we provide a technical discussion of our main results, first focusing on the scope and utility of our new rescaling formulas, and then turning to their accuracy and robustness. A graphical summary of all the rescaling formulas derived in the paper is presented in Fig. S3.

*Scope and utility of rescaling formulas*

Our first results in formulas (3) and (4) constitute a generalization of the rescaling derived for the contiguous SAR by Rosindell & Cornell (2007). These rescalings apply more generally to SARs generated by any pattern of habitat loss (Appendix 4). For a given clearing pattern, these rescalings reduce by one the effective number of parameters of the SAR by measuring space in units corresponding to the standard deviation of dispersal distance. The results serve two main purposes: (i) by reducing the number of parameters of an SAR, they simplify any analytical or numerical investigations into SAR properties; (ii) they serve as building blocks that can link different SAR formulas together.

Our second new class of SAR rescalings in formulas (5)–(7) show how, in certain limits, clumped patterns of clearing can produce approximately equal species loss to the more tractable cases of contiguous and random clearing. If the dispersal distance is large, the spatial structure in an clumped-clearing pattern, relative to a random-clearing pattern, is largely irrelevant, and the two collapse onto each other (Fig. 3; Fig. S1). On the other hand, if the dispersal distance is very small, each clump will be a nearly independent sample of the landscape, and the SAR can therefore be expressed as the sum of contiguous SARs. In practice, we expect the clumped-to-random rescaling formula (7) to be particularly useful because it is valid within the second phase of the SAR, which corresponds to a regional scale relevant to many applied species loss problems.

Our third class of SAR rescalings in formulas (8) and (9) is perhaps the most useful. These rescalings define an effective speciation rate, enabling us to relate the random-clearing SAR to a contiguous SAR. Thus, any knowledge about the well-studied contiguous SAR can be leveraged to understand the random SAR too. And, because contiguous and random clearing are the extreme cases of maximum and minimum spatial autocorrelation in clearing patterns, these two SARS provide upper and lower bound estimates of species loss in more general fragmented-clearing scenarios.

*Accuracy and robustness of rescaling formulas*

We used simulations to validate the final random-clearing formula (10) and found it to be highly accurate (Fig. S2): the average error was 1.2% or less depending on the parameterization, and this error was largely attributable to error in the Preston function approximation used (Eq. (S1)) and to the zero-sum nature of our simulations versus the non-zero-sum nature of the formula, rather than to error in our new rescaling relationship per se (formula (8); as evidenced by similar errors on the order of 1% in the contiguous-clearing formula; Fig. S2).

The rescaling relationship between random and contiguous SARs in formula (8) should be robust to moderate deviations from the idealized random-clearing pattern shown in Fig. 1D because it is based on the assumption that the probability of coalescence is roughly constant throughout the landscape. If habitat fragmentation is not truly random but clustered at a scale much smaller than the average species range, then the same result should continue apply.


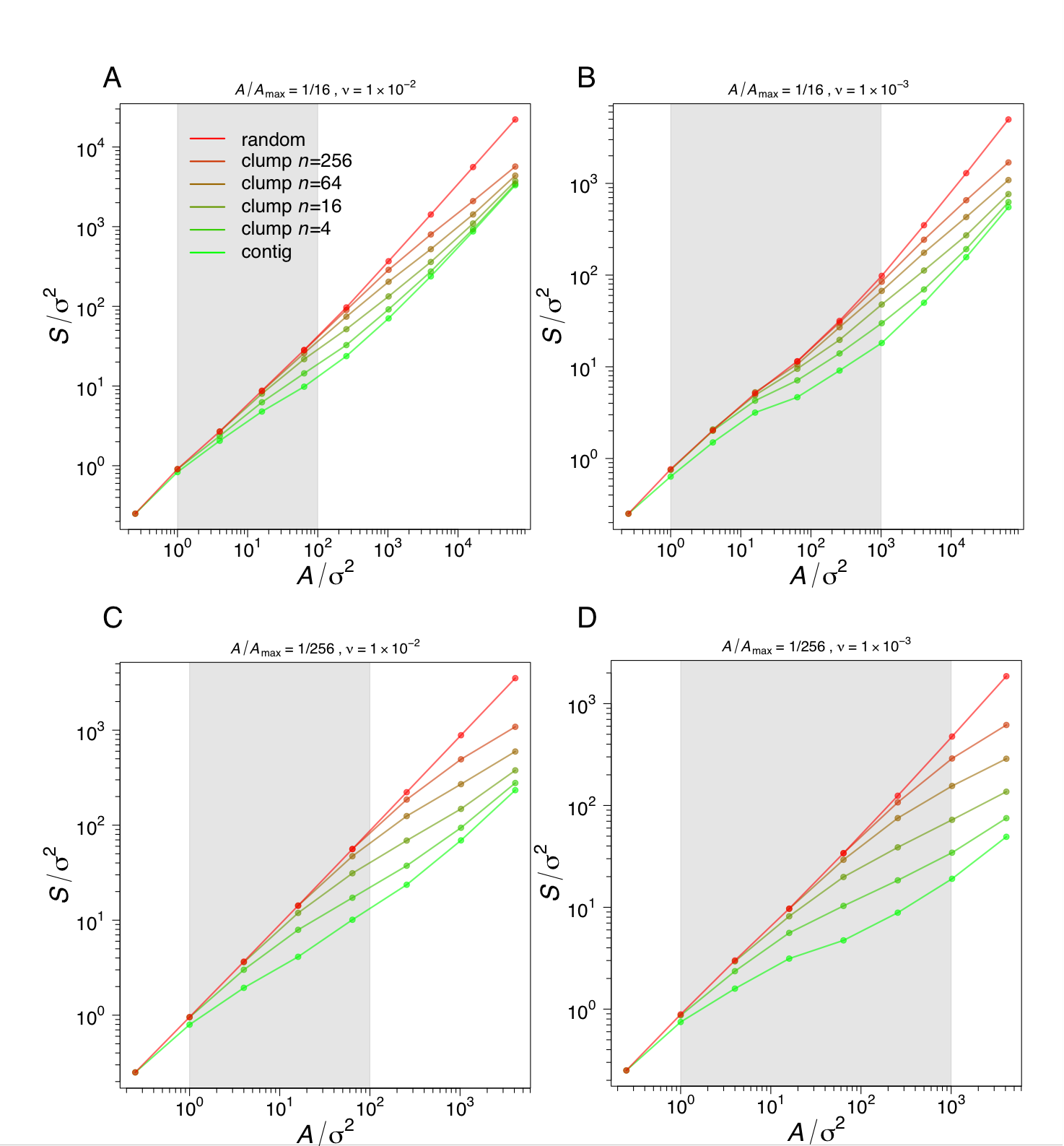


*Fig. S1*. As for Fig. 3 but with a broader range of values for the number of clumps $n$ in clumped clearing.


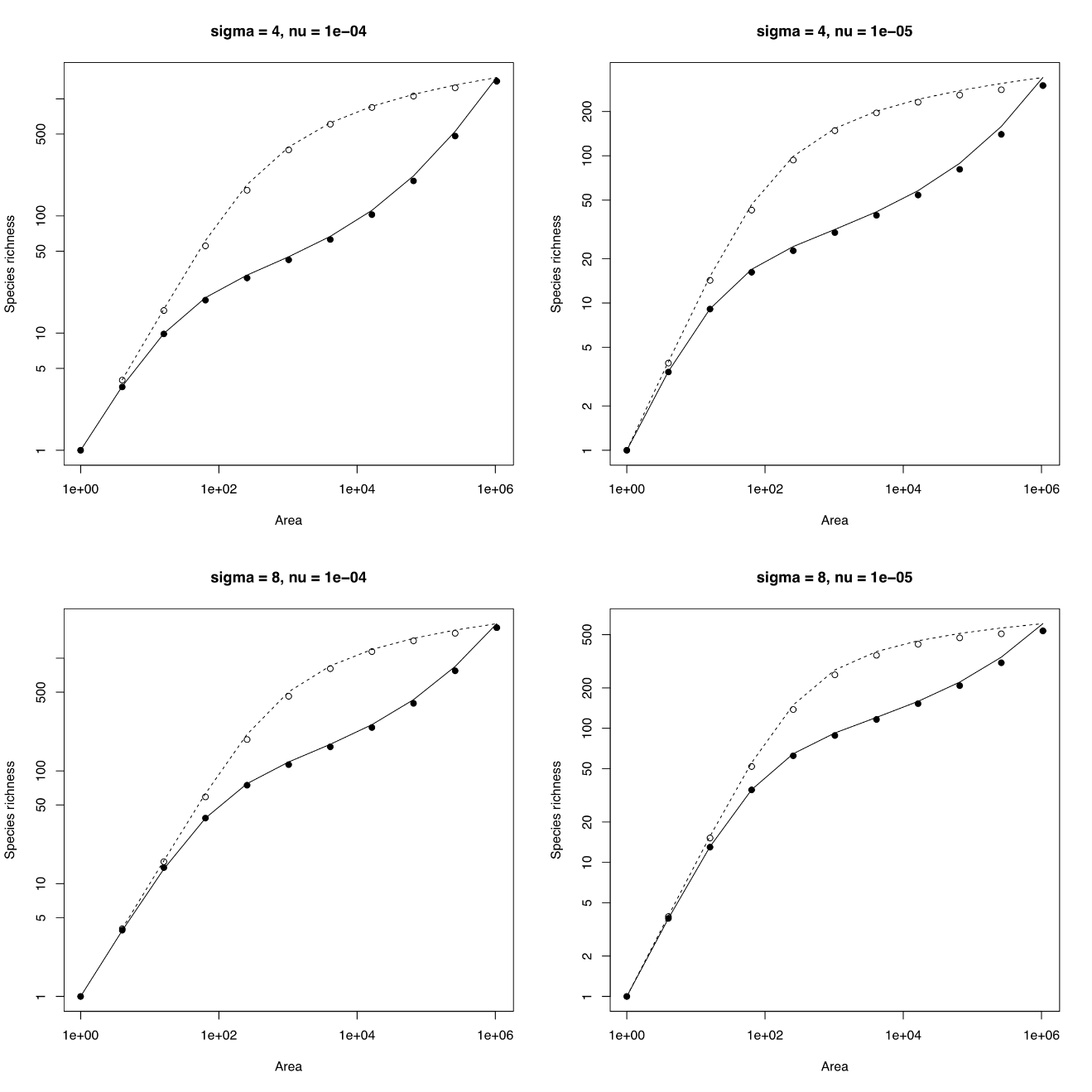


*Fig. S2.* Validation of the random SAR formula (formula (10)) for $A_{max}=2^{20}$ and different values of $\sigma$ and $\nu$ (panel titles). Dashed curves show values from formula (10); open circles show means of 100 simulations. For completeness, we also validate the contiguous SAR formula (solid curves and solid points), though this was already done by O’Dwyer & Cornell (2017). For the parameter values tested, mean absolute errors range from 0.6–1.2% for random clearing and 0.8–1.1% for contiguous clearing.


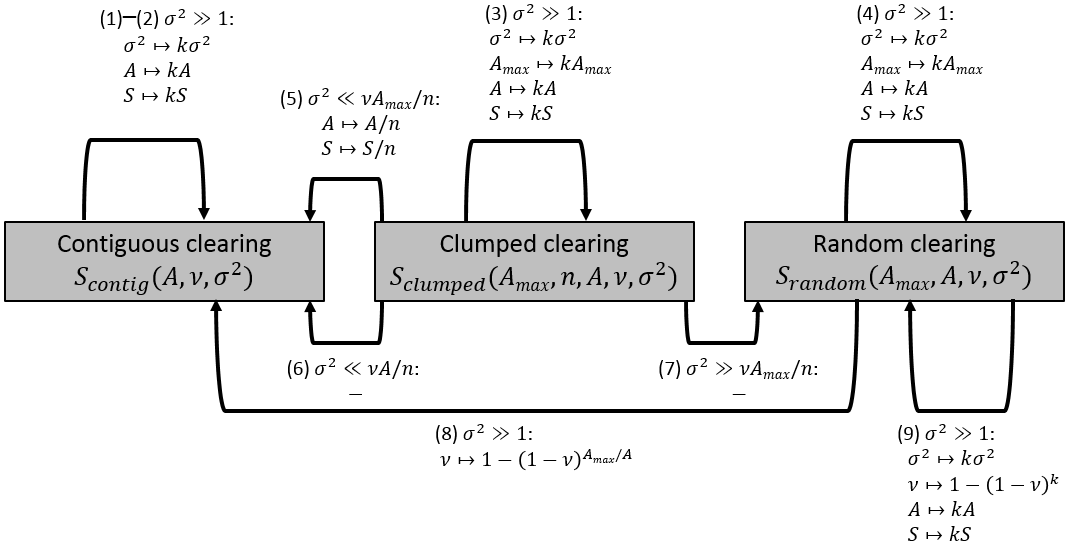


*Figure S3.* Summary diagram of SAR rescaling relationships numbered (1)–(9) derived in this paper, with each number corresponding to a formula in the main text. Thick arrows between boxes indicate the direction of a rescaling relationship. For each rescaling relationship, the condition on the dispersal parameter $\sigma^{2}$ under which the relationship holds is first given, and below it the parameter rescalings expressed with the “↦” symbol. Any of the SAR rescaling relationships can be reversed simply by reversing each corresponding parameter rescaling.

**Appendix 8: Further exploration of case studies**

We explored the sensitivity of the results for Barro Colorado Island (Fig. 6B) to different levels of habitat clearing ($A_{max}-A$) in the range 10–90%. For random clearing, we simply removed trees at random; for contiguous clearing, we chose a random starting point within the plot and progressively removed the trees furthest from this point until the clearing target was reached. We averaged species loss over 10,000 repeats of each simulation (this was sufficient for the standard error to be negligible). The results are shown in Fig. S4. The contiguous and random clearing formulas provide lower and upper bounds on species richness and, correspondingly, upper and lower bounds on species loss. The spatial-census-based estimates are intermediate to the formula estimates (Fig. S4), and the results are qualitatively consistent across values of area ($A$).


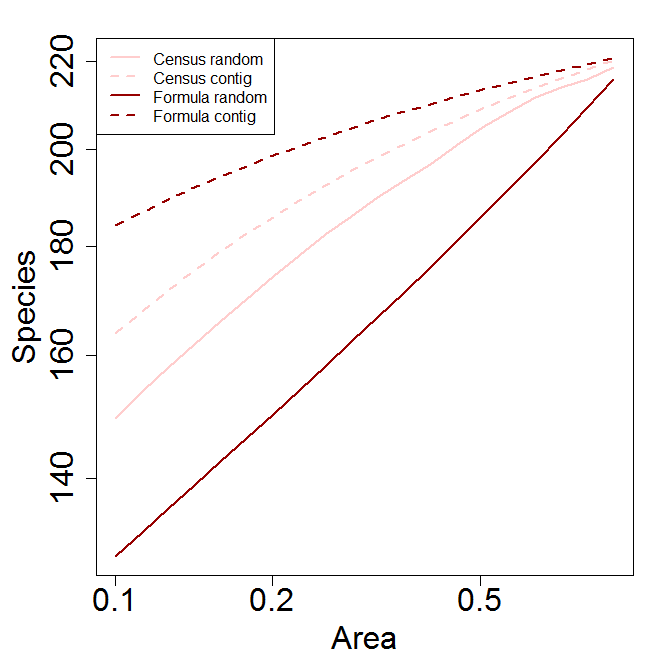


*Fig. S4.* Species–area curves for the BCI 50 ha plot. Dashed curves show random clearing; solid curves show contiguous clearing. The dark red curves are the theoretical bounds from our formulas (Eq. (S1) and formula (8)) and the pink curves are from simulated clearing on the actual spatial census data (averages of 10,000 repeats).

**References**

1.

Chave J. & Leigh E.G.J. (2002). A spatially explicit neutral model of ß-diversity in tropical forests. *Theor. Popul. Biol.*, 62, 153-168.

2.

Chisholm R.A., Fung T., Chimalakonda D. & O'Dwyer J.P. (2016). Maintenance of biodiversity on islands. *Proceedings of the Royal Society B: Biological Sciences*, 283.

3.

Condit R., Pitman N., Leigh E.G., Chave J., Terborgh J., Foster R.B., Nunez P., Aguilar S., Valencia R., Villa G., Muller-Landau H.C., Losos E. & Hubbell S.P. (2002). Beta-diversity in tropical forest trees. *Science*, 295, 666-669.

4.

Fisher R.A., Corbet A.S. & Williams C.B. (1943). The relation between the number of species and the number of individuals in a random sample of an animal population. *J. Anim. Ecol.*, 12, 42-58.

5.

Green J.L. & Ostling A. (2003). Endemics-area relationships: The influence of species dominance and spatial aggregation. *Ecology*, 84, 3090-3097.

6.

He F.L. & Legendre P. (2002). Species diversity patterns derived from species-area models. *Ecology*, 83, 1185-1198.

7.

Marvin D.C., Koh L.P., Lynam A.J., Wich S., Davies A.B., Krishnamurthy R., Stokes E., Starkey R. & Asner G.P. (2016). Integrating technologies for scalable ecology and conservation. *Global Ecology and Conservation*, 7, 262-275.

8.

Muller-Landau H.C., Wright S.J., Calderon O., Condit R. & Hubbell S.P. (2008). Interspecific variation in primary seed dispersal in a tropical forest. *Journal Of Ecology*, 96, 653-667.

9.

O'Dwyer J.P. & Cornell S. (2017). Cross-scale ecological theory sheds light on the maintenance of biodiversity. <https://arxiv.org/abs/1705.07856>.

10.

O'Dwyer J.P. & Green J.L. (2010). Field theory for biogeography: a spatially explicit model for predicting patterns of biodiversity. *Ecol. Lett.*, 13, 87-95.

11.

Rosindell J. & Cornell S.J. (2007). Species-area relationships from a spatially explicit neutral model in an infinite landscape. *Ecol. Lett.*, 10, 586-595.

12.

Rosindell J., Wong Y. & Etienne R.S. (2008). A coalescence approach to spatial neutral ecology. *Ecological Informatics*, 3, 259-271.
